# A Polymer Asymmetric Mach–Zehnder Interferometer Sensor Model Based on Electrode Thermal Writing Waveguide Technology

**DOI:** 10.3390/mi10100628

**Published:** 2019-09-20

**Authors:** Baizhu Lin, Yunji Yi, Yue Cao, Jiawen Lv, Yue Yang, Fei Wang, Xiaoqiang Sun, Daming Zhang

**Affiliations:** State Key Laboratory on Integrated Optoelectronics, College of Electronic Science and Engineering, Jilin University, Changchun 130012, China; linbz17@mails.jlu.edu.cn (B.L.); yiyj@jlu.edu.cn (Y.Y.); yuecao17@mails.jlu.edu.cn (Y.C.); lvjw18@mails.jlu.edu.cn (J.L.); a2604702999@163.com (Y.Y.); wang_fei@jlu.edu.cn (F.W.); sunxq@jlu.edu.cn (X.S.)

**Keywords:** polymer waveguides, integrated optics, sensors, thermal writing waveguide

## Abstract

This paper presents a novel electrode thermal writing waveguide based on a heating-induced refractive index change mechanism. The mode condition and the electrode thermal writing parameters were optimized, and the output patterns of the optical field were obtained in a series of simulations. Moreover, the effect of various adjustments on the sensing range of the nanoimprint M–Z temperature sensor was analyzed theoretically. A refractive index asymmetry Mach–Zehnder (M–Z) waveguide sensor with a tunable refractive index for a waveguide core layer was simulated with a length difference of 946.1 µm. The optimal width and height of the invert ridge waveguide were 2 μm and 2.8 μm, respectively, while the slab thickness was 1.2 μm. The sensing accuracy was calculated to range from 2.0896 × 10^4^ to 5.1252 × 10^4^ in the 1.51–1.54 region. The sensing fade issue can be resolved by changing the waveguide core refractive index to 0.001 via an electrode thermal writing method. Thermal writing a single M–Z waveguide arm changes its refractive index by 0.03. The sensor’s accuracy can be improved 1.5 times by the proposed method. The sensor described in this paper shows great prospects in organism temperature detection, molecular analysis, and biotechnology applications.

## 1. Introduction

Rapid advancements of the Internet of Things (IoT) have made wearable devices a subject of increasing research and development interest. Any wearable device functions based on its sensors. For example, a multisensory electronic skin can be used to simultaneously monitor various parameters such as temperature, strain, pressure, and magnetic field characteristics [1]. An optical signal is an ideal information transmission medium due to its fast transmission rate, small loss, and large capacity. Polymer organic materials have good biocompatibility among the materials available. They are applicable in high-precision and low-cost processing techniques such as laser writing and nanoimprint [2,3]. Polymer photonic skin has broad prospects in the fields of information transmission, sensing, and human–computer interaction.

Existing polymer optical sensors mainly include the metal surface plasmon waveguide sensor [4,5,6], the Mach–Zehnder (M–Z) waveguide sensor [7,8], and the optical fiber stress sensor [9,10]. Currently, sensing temperature [11,12,13,14] and antibody antigens [15,16,17] is widespread in the organism-sensing field. Many optical sensors are capable of these functions, of which the majority are based on M–Z optical waveguide. Mach–Zehnder interferometers (MZIs) are often used as biosensors in chemical and biological applications via utilizing the method of critical evanescent field detection [18]. MZI biosensors are label free, non-destructive, open to simple on-chip integration, and do not require spectral detection equipment.

Previous researchers have established an MZI based on SU-8 resist as the core/cladding waveguide material for biochemical detections with a detection sensitivity of 10^−12^ g/mL (for sheep anti-IgG (Immunoglobin G)) [19]. Another MZI exhibited a spatial resolution of about 10 mm and a detection limit down to 10^−4^ RIU [20]. Despite its multiple advantages, the MZI device has the issue of aligning the waveguide with a microfluidic channel. Asymmetrical M–Z structures have been designed in an effort to remedy the alignment issue [21,22]. In 2016, an asymmetric MZI inverted ridge waveguide sensor based on a polymer substrate was designed and fabricated. The prototype showed a detection limit of 3 × 10^−3^ refractive index units for homogeneous sensing at a total system length of 9.3 mm and a total waveguide core thickness of 3 μm [23]. In 2017, a polymer-based effective index sensor based on tailored high-index thin films was reported to enhance the sensitivity of asymmetric MZIs [24]. The core layer of this M–Z structure was fabricated with a single material, resulting that the phase difference of the two waveguide arms can only be realized by changing the length and width of the waveguide. However, the refractive index is difficult to adjust. Hybrid silicon/SU-8 and silicon/TiO_2_ waveguides have also been employed to replace one arm of the conventional all-silicon MZI waveguide, of which the temperature sensitivities are 172 pm/°C and 775 pm/°C, respectively, and the resolution can reach to 6 × 10^−3^ °C [25]. These structures require multiple alignment and etching windows, which makes the fabrication process tedious.

In this study, we developed a novel waveguide fabrication method based on electrode thermal writing, which allows for tuning the refractive index of the core layer with the same material. By fine-tuning the width and heating temperature of the electrode, the waveguide with an adjustable refractive index for the core layer was fabricated by electrode thermal writing. The refractive index of material can be altered via applying a thermal field so that waveguide structures with different refractive indices can be obtained on the same chip. According to this theory, we further resolve the sensing fade issue and increase the sensing accuracy of a simulated sensor by tuning the refractive index of an asymmetrical MZI core layer.

## 2. Theoretical Analysis

**Figure 1:**
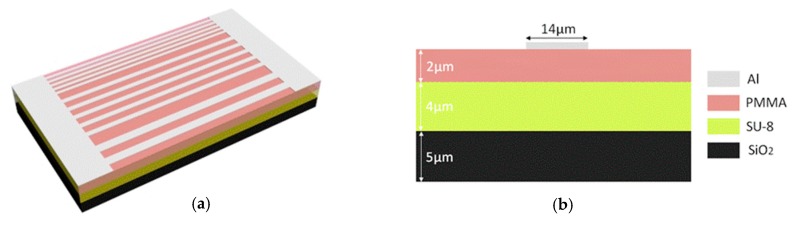
(**a**) Schematic of the electrode thermal writing waveguide and (**b**) cross-sectional view of the waveguide.

Figure 1a shows the schematic of the electrode thermal writing waveguide. A cross-sectional view of the waveguide is shown in Figure 1b. An SiO_2_ layer was selected as the substrate. The refractive index and thermal conductivity of SiO_2_ are 1.444 and 1.4 W/(m·K), respectively. SU-8 2005 was selected as the core layer, which is easily fabricated, and the refractive index can be tuned by the temperature at which the electrode is written. Its refractive index and thermal conductivity are 1.571 and 0.2 W/(m·K), respectively. PMMA (Polymethyl Methacrylate) was selected as the polymer upper cladding material, in which the refractive index and thermal conductivity are 1.4783 and 0.19 W/(m·K), respectively. Strip aluminum electrodes with different widths from 6 µm to 14 µm were fabricated. Once the electrode was thermally written, different heating effects could be obtained with the same electrical power.

**Figure 2:**
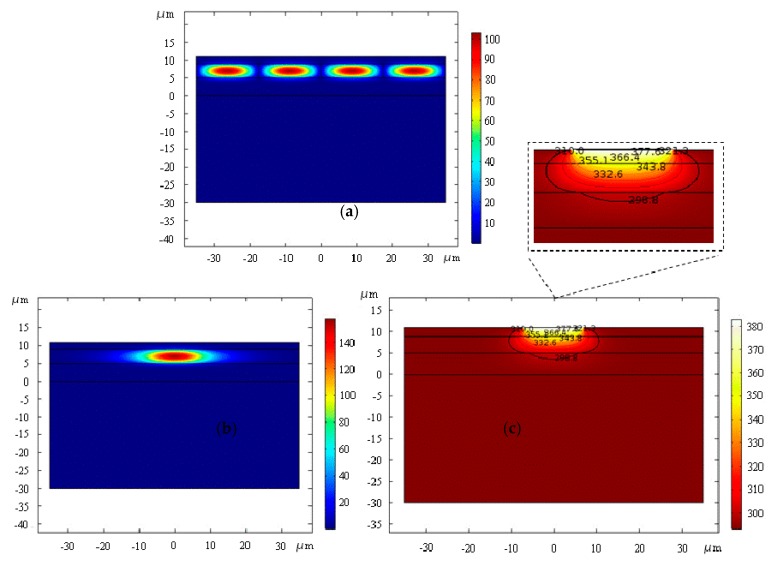
(**a**) Optical field distribution before and (**b**) after applying electric power and (**c**) thermal field distribution of the electrode thermal writing waveguide.

The optical field distribution of the device with and without applying electric power are shown in Figure 2. When no electric power was applied to the device, the structure of the waveguide was a plate, and the optical field was limited to the whole core layer, producing a row of plate mode effects (Figure 2a). After applying electric power to the device, the thermal field induced by the strip electrode would cause an irreversible change of the refractive index of the core layer, and the waveguide will be written into the core layer according to the strip electrode position. The thermal field was simulated with the electrode width of 14 μm, as shown in Figure 2c. The functional relationship between the refractive index and temperature of SU-8 was fitted using the formula as follows [26]:

(1) $$RI=5.631\cdot 10^{-9}\cdot \Delta T^{3}-1.006\cdot 10^{-6}\cdot \Delta T^{2}+4.797\cdot 10^{-5}\cdot \Delta T+1.575$$

where *RI* is the refractive index of SU-8, and Δ*T* is the temperature acting on the SU-8.

When simulating the optical field of the device, the function relation was brought into the change of refractive index of SU-8 with temperature. The mode of the thermal writing waveguide depends on the thickness of the upper layer and the temperature at which the electrode is written. Due to the existence of an upper layer, a gradient change exists in the thermal field when reaching the core layer. Therefore, by controlling the temperature, the width of the electrode, and the thickness of the upper layer, the mode can be tuned, and the single-mode transmission can be achieved.

The influence of different electrode widths on the waveguide mode is analyzed. When the electrode width increases within the effective range, the leakage of a single mode to both sides keeps declining according to the Finite Element Method (FEM)—that is to say, the limit is getting better. So, the electrode width of 14 μm was selected to analyze the optical field and thermal field, as well as the mode field distribution after thermal writing. When the temperature increases by 90 K, the optical field of the waveguide is shown in Figure 2b, showing a single mode effect. Figure 2c exhibits the thermal field of the waveguide.

The electrode thermal writing waveguides were fabricated by the conventional photolithography and wet-etching method. First, SU-8 was spin-coated on the substrate at a speed of 6000 r/min for 20 s, obtaining a film with the thickness of 4 μm. After baking, PMMA was then spin-coated on the sample and baked at 120 °C for 20 min (MODELKW-4AH, Hexin Corporation, China) to form a 2 μm thick upper cladding. Finally, Al electrode heaters with the thickness of 0.1 μm were deposited on the upper cladding by thermal evaporation (DM-300B, Gujie Corporation, China), photolithography, and wet-etching processes. The width of the strip aluminum electrode was 6–14 μm here. In order to apply electric power, a width of about 3 mm was reserved at both ends of the electrode.

**Figure 3:**
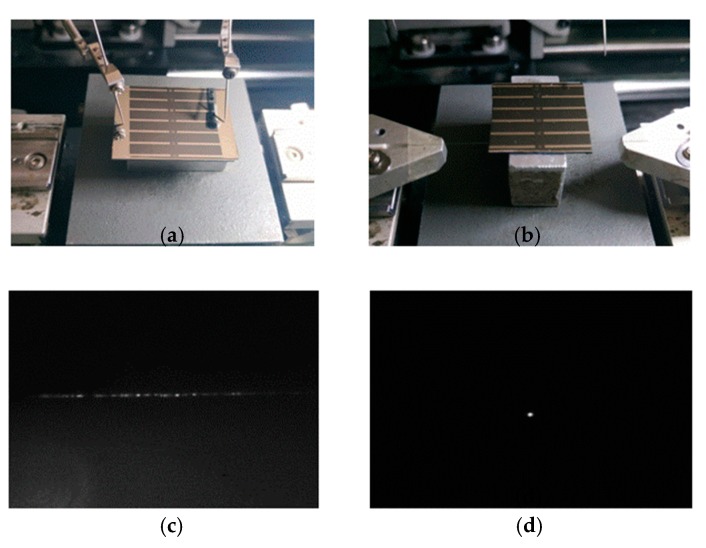
(**a**) Measuring process of applying electric power; (**b**); the testing photo of the TO switch with electric power applied; (**c**) Relative output patterns before power applied and (**d**) output patterns after the electric power applied.

The output patterns of a strip electrode structure with and without applying electric power at 1550 nm were compared, and the width of the electrode is 14 μm. Figure 3a presents a test photo of applying electric power. After the end face of the device was cleaved without applying electric power, the test setup was shown in Figure 3b. The fabricated electrode thermal writing waveguide was characterized by optical transmission measurements. A light beam (at 1550 nm) generated by a solid-state laser was directly coupled into the input port of the device through a customized small-diameter polymer optical fiber. The output light was detected using a photodetector and measured by a power meter. For the optical measurement, the output light from the device was firstly focused using a microscope objective lens that images the output patterns, and captured by a charge-coupled device (CCD) camera. When no electric power was applied to the device, the relative patterns were displayed on a video monitor, as shown in Figure 3c. There was a row of plate mode effects due to the plate structure of the waveguide. When the electric power was applied to the device, the relative output patterns of the device with the length of 2 cm were observed, as shown in Figure 3d. To measure the insertion loss of the device, the light beam was coupled directly into the optical power meter by a similar polymer optical fiber. The insertion loss was obtained to be less than 15 dB (end surface not polished), including the coupling loss, transmission loss, and excess loss. When the applied electric power gradually increased to 20 V for 10 min, the output patterns of the device can be seen. The resistance of the Al electrode is about 12 Ω, so the heating power is 33.3 W. The output patterns did not change when the electric power was turned down, which proved the irreversibility of the electrode thermal writing method.

After the electrode thermal writing waveguide was tested and the relative output patterns were obtained, the effect of various adjustments on the sensing range of the nanoimprint M–Z temperature sensor was analyzed. First, we studied an electrode thermal writing optical waveguide that was compatible with the printing technology. Then, the relationship between waveguide mode and electrode temperature were analyzed. Furthermore, the effects of different heat-written temperatures of the two arms of the polymer M–Z temperature sensor on the performance of the device were investigated.

## 3. Design and Simulation

**Figure 4:**
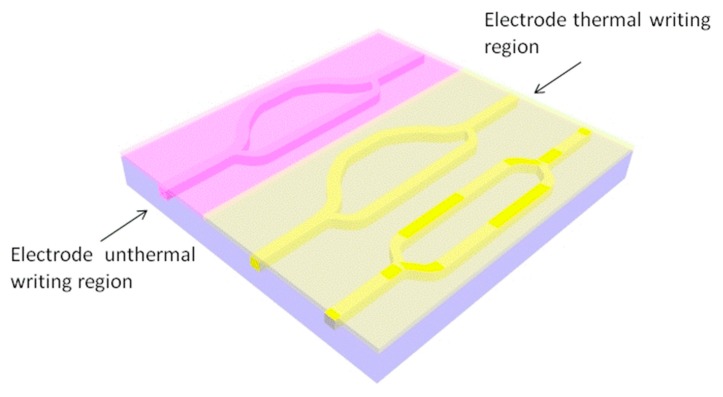
The schematic of the asymmetric Mach–Zehnder (M–Z) waveguide arrays.

We designed M–Z sensor arrays composed of three asymmetric M–Z waveguide sensors: a low-accuracy M–Z sensor with one monotonous sensing range, and two high-accuracy M–Z sensors with several monotonous sensing ranges. The low-precision monotonic sensor was designed to determine the high precision sensor’s sensing interval, while the high-precision sensing enhanced the sensor’s accuracy. To this effect, the electrode thermal writing method provided another opportunity to adjust the high-precision sensor’s range while impeding any sensing degeneration in the M–Z device. A schematic diagram of the M–Z sensor arrays is provided in Figure 4.

**Figure 5:**
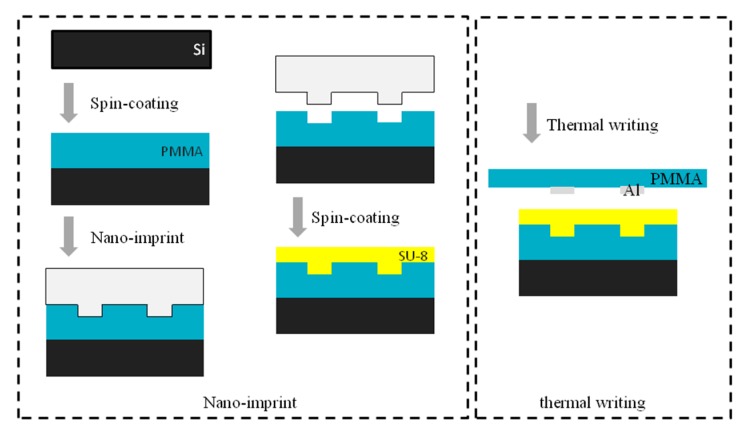
Fabrication process of the waveguide by nanoimprint and schematic diagram of thermal writing.

The fabrication process of the waveguide by nanoimprint and the schematic diagram of thermal writing is shown in Figure 5. The design of the device is based on the polymer SU-8, which can be easily mass-produced, has favorable mechanical properties, and has a refractive index that can be adjusted by electrode thermal writing. PMMA was adopted as the lower cladding material, which is compatible with the nanoimprint method. A 1550 nm laser, which is compatible with the optical communication wavelength, was selected as a light source. To maximize the effect of electrode thermal writing and minimize the cost, a nanoimprint was used instead of wet etching. The inverted ribbed waveguide based on this technique is the most compatible waveguide type. The electrode is fabricated on the polymer substrate PMMA, which has transparent characteristics that are convenient for waveguide alignment, and the width of the electrode is slightly larger than that of the waveguide. Two externally connected leads are fabricated at the ends of the electrode to connect voltage, and the designated areas are heated by alignment in an inverted and fitted manner. So, the surface layer can be kept bare, and the electrode does not need to be removed and can be repeatedly utilized.

There are three waveguide design factors to consider before fabricating asymmetrical M–Z waveguide sensors: waveguide sensitivity, single-mode condition, and waveguide loss. Sensitivity is an important criterion of sensor performance. It is generally defined as the sensitivity of the symmetric MZI sensor’s refractive index; the sensitivity of the asymmetric MZI structure can be determined by the structural parameters of the waveguide, and can be optimized as per the design of the waveguide’s cross-section.

Core thickness (*H*), groove depth (*d*), and width (*w*) are the main design parameters of the inverted ribbed waveguide structure. Restricting factors are insertion loss, single-mode condition, and waveguide sensitivity. A single-mode transition should be ensured to avoid ambiguous signals. According to the first rank mode cut-off thickness formula of an inverted ribbed waveguide structure, the designed structure can achieve superior single mode propagation. The finite element difference results were simulated to test this superiority with w and d values of 2 μm and 2.8 μm, respectively. In this setup, in the case of a lower cladding refractive index where *n*_s_ = 1.49, the refractive index of the core layer is *n*_g_ = 1.571, the upper cladding refractive index is *n*_c_ = 1.49, and the operating wavelength is 1550 nm.

**Figure 6:**
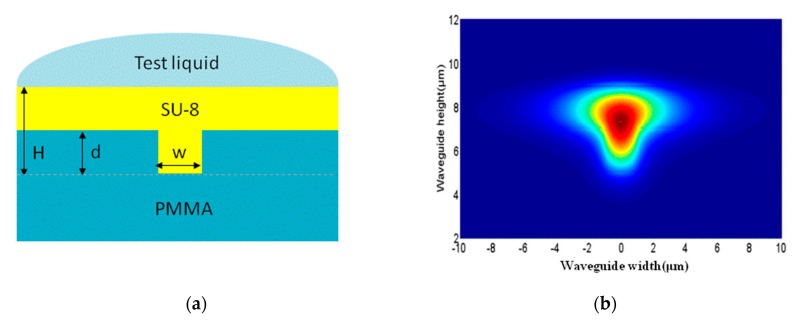
(**a**) Cross-section and (**b**) optical field distribution of inverted ridge waveguide.

Mismatch between the mode fields of the ribbed waveguides and Gaussian distributed fibers can cause an end-coupling loss, which is the main insertion loss of the entire device. By calculating the end-coupling coefficient, we concluded that a thicker core layer results in a smaller coupling loss. We also determined that waveguide sensitivity increases as the core layer thickness decreases, which creates another problem. Taking both phenomena into consideration, a 4-μm thickness is optimal. The cross-section of the inverted ridge waveguide is shown in Figure 6a, and the optical field distribution is shown in Figure 6b.

**Figure 7:**
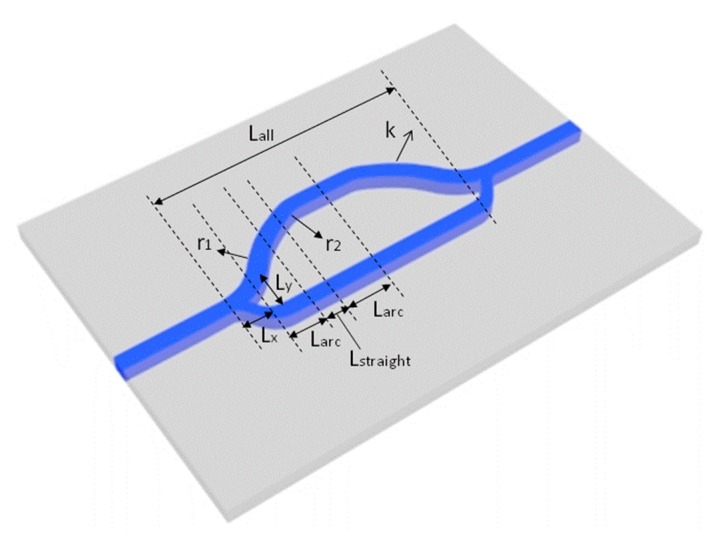
The parameters schematic of the Asymmetrical Mach–Zehnder Interferometer (AMZI) waveguide sensor with high sensing accuracy.

We simulated the bending loss of the designed inverted ribbed waveguide to complete the design of the MZI device. The bending radius was optimal at 3000 µm. The MZI structure is based on two main parameters: the difference length of two arms (Δ*L*) and the effective refractive index of layers (Neff), which determine the sensing range and the highest-accuracy sensing point. The optimal asymmetrical M–Z structure parameters we identified are shown in Figure 7. A straight waveguide and a bent waveguide were designed to increase the length difference of the two M–Z arms. Among them, the long arm is composed of two bent waveguides with the bending radius of *r*_1_ and *r*_2_, and a straight waveguide with a transverse length of *L*_straight_. *k* is the slope of the relative propagation axis of the straight waveguide. The transverse length of the bent waveguide is *L*_arc_; it is a function of curvature radius *r* and *k*, while *L*_x_ and *L*_y_ are the length and width of the Y branch, respectively. According to the arc-type S-bend structure we designed, the length difference between the sensing arm (long arm) and the reference arm (short arm) is given by the following formula:

The length difference was optimized to 25.5–946.1 μm. The parameters of asymmetrical Mach–Zehnder Interferometer (AMZI) sensors with different length differences are listed in Table 1.

(2) $$\Delta L=L_{s}-L_{r}$$

(3) $$L_{i}=4L_{\mathrm{arci}}+2(k_{i}{}^{2}L_{\mathrm{straighti}}{}^{2}+L_{\mathrm{straighti}}{}^{2}{)}^{\frac{1}{2}},i=\{\begin{array}{c} s,\begin{array}{ll} sen\sin g & arm \end{array}\\ r,\begin{array}{cc} reference & arm \end{array} \end{array}$$

**Figure 8:**
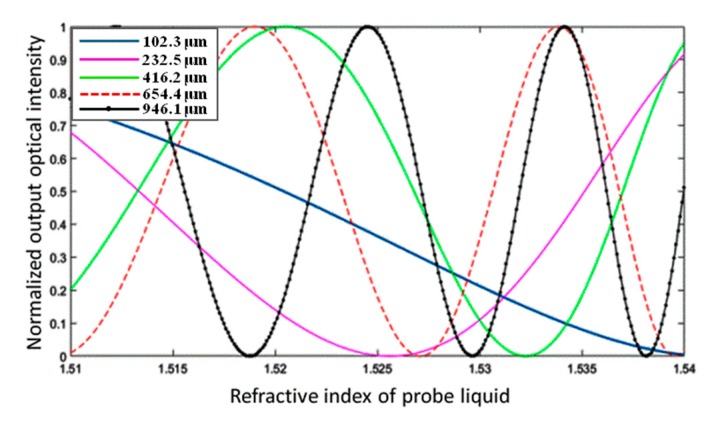
Normalized output optical intensity with various sensing arm length differences.

The transmitted intensity with a variety of length differences is shown in Figure 8. The M–Z device with a 102.3 µm length difference has a sensing range from 1.51 to 1.54. The M–Z device with a 946.1 μm length difference has a 5.5-fold increase in sensing accuracy compared to the device with a 102.3 μm length difference. The various sensing ranges of the M–Z device can be confirmed by using an M–Z device with a 102.3 μm arm length difference.

**Figure 9:**
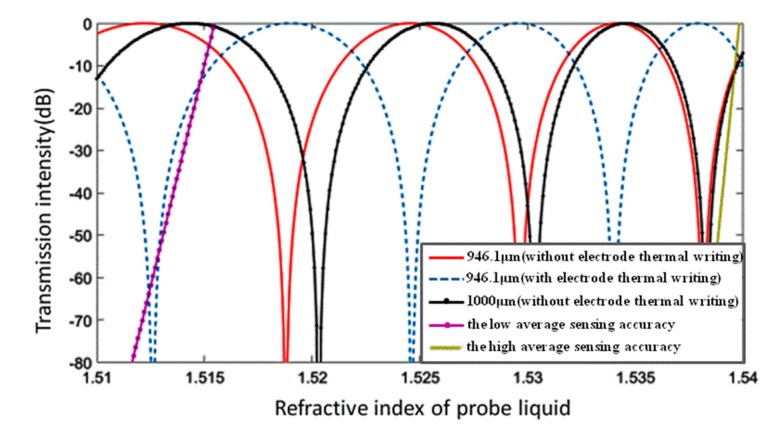
Transmission intensity of sensor with various length differences and core refractive indices.

There is a trade-off between the sensing accuracy and range of M–Z interferometers because of its basic working principles. The sensing region fades quickly as the sensitivity increases. M–Z waveguide arrays may be used to resolve the sensing fade problem by changing the length difference of the asymmetrical M–Z waveguide sensors, thus distributing the sensing range across a larger region. However, the output curve distribution of this kind of waveguide sensor array was not distributed perfectly in our setup, as shown in Figure 9. The devices with length differences of 946.1 μm and 1000 μm have complementary sensing ranges in the low refractive index region, but said sensing ranges nearly coincide in the high refractive index region. We attempted to solve the sensing fade problem by introducing another M–Z structure with electrode thermal writing technology and a 946.1 μm length difference.

The electrode thermal writing method can be used to fine-tune the core refractive index. As the core refractive index is adjusted, the output curve distribution of the sensor can be regulated in the whole sensing region. This produces a larger sensing range via two asymmetric M–Z waveguide sensors with different writing temperatures. The sensing range of the high-accuracy M–Z sensors (Δ*L* = 946.1 µm) is determined by the low sensing accuracy waveguide sensor (Δ*L* = 102.3 µm). This reflects the trade-off between sensing accuracy and sensing range. By adjusting the refractive index of the photosensitive polymer core waveguide of the two high sensing M–Z waveguide sensors via electrode thermal writing, we mitigated the M–Z waveguide sensor fading problem. The output power values with different core refractive indices are shown in Figure 9. The average sensing accuracy of our M–Z waveguide array falls between 2.0896 × 10^4^ and 5.1252 × 10^4^ in the range of 1.51 to 1.54.

**Figure 10:**
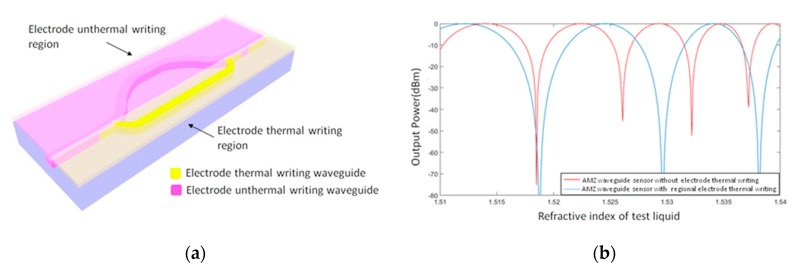
Schematics (**a**) and output power (**b**) of local electrode thermal writing device.

**Figure 11:**
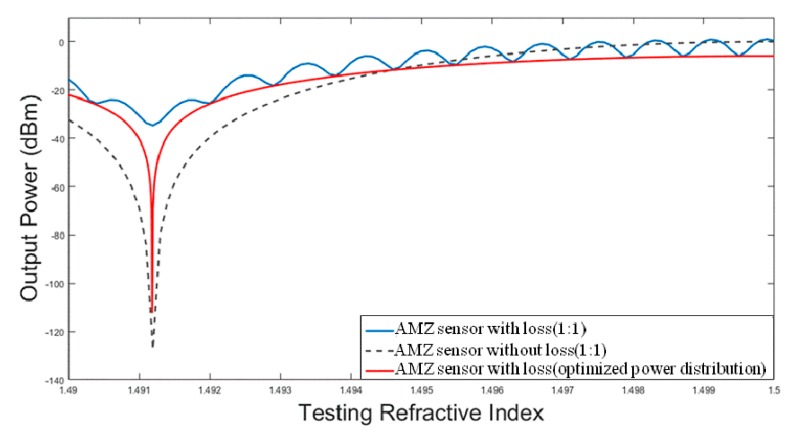
The output power of AMZ sensor with an optimized Y-splitter power (with loss of 2.5 dB/cm, Δ*L* = 946.1 µm).

Electrode thermal writing in a single AMZI device also increases the accuracy of the sensor. A schematic diagram of adjustments to the refractive index of one arm of the waveguide by means of electrode thermal writing is shown in Figure 10a; the output power of the sensor is shown in Figure 10b. The sensitivity of the sensor (Δ*L* = 946.1 µm) can be increased 1.5-fold in the high refractive index region (1.53–1.538). For the asymmetric waveguide sensor, the output power was affected by the loss difference of the two branches. This is likely because the loss of the polymer waveguide cannot be neglected (0.01–3 dB/cm). The output power can be expressed by the expression:

(4) $$I_{T}=A_{1}e^{\alpha L_{1}}(\cos N_{1}\frac{2\pi }{\lambda }L_{1})+\sin(N_{2}\frac{2\pi }{\lambda }L_{1})i+A_{2}e^{\alpha L_{2}}(\cos N_{2}\frac{2\pi }{\lambda }L_{2})+\sin(N_{2}\frac{2\pi }{\lambda }L_{2})i$$

where *I_T_* and *α* are the sensor’s output power and the waveguide loss factor, respectively. *L* is the length of the arm, *N* is the effective refractive index, and *A* is the power of each arm (subscripts 1 and 2 denote two different arms, respectively). The output power of the device fluctuates with a symmetric Y-splitter, as shown in Figure 11. In order to balance the optical power of the two sensing arms, we optimize the Y-splitter power distribution to 0.56:0.44. The Y-splitter power distribution can be expressed by the expression: $\frac{1}{1+e^{\left(\alpha \cdot \Delta L\right)}}:\frac{e^{\left(\alpha \cdot \Delta L\right)}}{1+e^{\left(\alpha \cdot \Delta L\right)}}$, where Δ*L* is length difference. Then, the smooth output characters can be determined.

## 4. Conclusions and Perspectives

This paper introduced an electrode thermal writing method for adjustable sensing range modalities in asymmetric waveguide sensor arrays. Sample electrode thermal writing M–Z waveguide arrays proved superior to length difference-based M–Z waveguide arrays with respect to the sensing range (especially the high refractive index region) and accuracy. With a length difference of 946.1 µm, the sensing accuracy was calculated to range from 2.0896 × 10^4^ to 5.1252 × 10^4^ in the 1.51–1.54 region. The effects of waveguide loss on the sensing characteristics were also analyzed with a loss of 2.5 dB/cm. The optimal power distribution was identified at 0.56:0.44.

In the future, we will analyze the bending loss and mode characteristics of the Y branch, and study the heating effect of the irregular electrode pattern and the influence on the refractive index. Also, we plan to develop MZI devices by thermal writing and apply an electrode thermal writing device in a high-index waveguide sensor to increase its sensing accuracy and sensing range.

## Figures and Tables

**Table 1:** Asymmetrical Mach–Zehnder Interferometer (AMZI) sensor parameters associated with various length differences.

*k*	*L*_straight_ (µm)	*L*_all_ (µm)	Δ*L* (µm)
0.3	9997	24591.6	946.1
0.25	9997	23874.6	654.4
0.2	9992	23121.9	416.2
0.15	9998	22369.4	232.5
0.1	9997	21566.1	102.3
0.05	10075	20949.0	25.5
